# Reliable brain morphometry from contrast‐enhanced T1w‐MRI in patients with multiple sclerosis

**DOI:** 10.1002/hbm.26117

**Published:** 2022-10-17

**Authors:** Michael Rebsamen, Richard McKinley, Piotr Radojewski, Maximilian Pistor, Christoph Friedli, Robert Hoepner, Anke Salmen, Andrew Chan, Mauricio Reyes, Franca Wagner, Roland Wiest, Christian Rummel

**Affiliations:** ^1^ Support Center for Advanced Neuroimaging (SCAN), University Institute of Diagnostic and Interventional Neuroradiology University of Bern, Inselspital, Bern University Hospital Bern Switzerland; ^2^ Graduate School for Cellular and Biomedical Sciences University of Bern Bern Switzerland; ^3^ Swiss Institute for Translational and Entrepreneurial Medicine Bern Switzerland; ^4^ Department of Neurology Inselspital, Bern University Hospital and University of Bern Bern Switzerland; ^5^ ARTORG Center for Biomedical Research University of Bern Bern Switzerland

**Keywords:** brain morphometry, cortical thickness, deep learning, MRI, post‐contrast imaging

## Abstract

Brain morphometry is usually based on non‐enhanced (pre‐contrast) T1‐weighted MRI. However, such dedicated protocols are sometimes missing in clinical examinations. Instead, an image with a contrast agent is often available. Existing tools such as FreeSurfer yield unreliable results when applied to contrast‐enhanced (CE) images. Consequently, these acquisitions are excluded from retrospective morphometry studies, which reduces the sample size. We hypothesize that deep learning (DL)‐based morphometry methods can extract morphometric measures also from contrast‐enhanced MRI. We have extended DL+DiReCT to cope with contrast‐enhanced MRI. Training data for our DL‐based model were enriched with non‐enhanced and CE image pairs from the same session. The segmentations were derived with FreeSurfer from the non‐enhanced image and used as ground truth for the coregistered CE image. A longitudinal dataset of patients with multiple sclerosis (MS), comprising relapsing remitting (RRMS) and primary progressive (PPMS) subgroups, was used for the evaluation. Global and regional cortical thickness derived from non‐enhanced and CE images were contrasted to results from FreeSurfer. Correlation coefficients of global mean cortical thickness between non‐enhanced and CE images were significantly larger with DL+DiReCT (*r* = 0.92) than with FreeSurfer (*r* = 0.75). When comparing the longitudinal atrophy rates between the two MS subgroups, the effect sizes between PPMS and RRMS were higher with DL+DiReCT both for non‐enhanced (*d* = −0.304) and CE images (*d* = −0.169) than for FreeSurfer (non‐enhanced *d* = −0.111, CE *d* = 0.085). In conclusion, brain morphometry can be derived reliably from contrast‐enhanced MRI using DL‐based morphometry tools, making additional cases available for analysis and potential future diagnostic morphometry tools.

## Graphical abstract

Brain morphometry can be derived reliably from contrast‐enhanced MRI using DL+DiReCT, a deep learning‐based morphometry tool. Making MR images acquired for clinical examinations with a contrast agent accessible for quantitative analysis is of interest for retrospective studies and potential future diagnostic support tools.

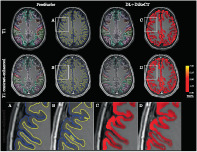

## 1 INTRODUCTION

Brain morphometry is usually derived from high‐resolution, 3D T1‐weighted (T1w) MRI, owing to the good gray/white‐matter contrast of protocols such as MP‐RAGE (van der Kouwe et al., 2008). Morphometry tools such as FreeSurfer (Fischl, 2012) usually expect non‐enhanced (pre‐contrast) MR images of this type as input data. For diagnostic purposes, T1w images after administration of gadolinium‐based contrast agents (GBCAs) are often acquired (Traboulsee et al., 2016), either as a replacement for or in addition to the non‐enhanced image. MRI acquired for clinical purposes sometimes include only contrast‐enhanced 3D T1w MRI, leaving those data inaccessible for retrospective morphometric analysis. For example, the latest MR imaging guidelines for multiple sclerosis recommend a contrast‐enhanced T1w acquisition, but not necessarily a corresponding image without contrast‐agent (Wattjes et al., 2021).

Few studies have investigated the influence of contrast agents on morphometry. A significant impact was reported on thalamic volumes (Hannoun et al., 2018) and tissue relaxation times (Warntjes et al., 2014). Good results using FreeSurfer were recently reported for volumes and thickness on a small and pre‐selected sample (Lie et al., 2022).

In multiple sclerosis (MS), quantification of the global gray‐matter (GM) loss is already used as a secondary outcome measure in clinical trials (Radü et al., 2013; Sastre‐Garriga et al., 2020). With the adoption of No evidence of disease activity (NEDA) in its updated version NEDA‐4 (Kappos et al., 2016), brain atrophy quantification has become increasingly important for the assessment of individual patients. For research purposes, studying the distribution of MS‐typical atrophy patterns over the cortex (like observable in regional GM volume or cortical thickness) in addition to the global GM volume might help improve the understanding of the disease (Eshaghi et al., 2018; Steenwijk et al., 2016). Beyond these univariate approaches, network properties of cortical reorganization due to aging or neurodegenerative processes can be studied by investigating coordinated structural changes of different subregions of the cortex using the concept of structural covariance networks (SCN; Alexander‐Bloch et al., 2013; Evans, 2013). In the context of MS, SCNs have been used to study the impact of this reorganization on physical and cognitive impairment (Tewarie et al., 2014), to reveal differences between conversion vs. non‐conversion from clinically isolated syndrome to MS (Tur et al., 2018), or to show early longitudinal reorganization processes in patients with relapsing remitting (Fleischer et al., 2019) or primary progressive MS (Tur et al., 2020) as compared to healthy controls. Importantly, none of these findings were noticeable in simpler univariate analyses of global or regional cortical thickness.

Cortical thickness is a frequently used measure in surface‐based morphometric analysis (Fischl & Dale, 2000), employed by many of the largest brain morphometry studies (Thompson et al., 2020). Being largely independent of head size (Schwarz et al., 2016) and less correlated to the corresponding volume measures (Winkler et al., 2010) makes it an interesting independent quantitative marker. Its self‐explanatory nature is another advantage for potential clinical applications.

The urgent need to make large amounts of additional T1w MRI accessible to morphometric analysis, particularly retrospective analysis of CE images acquired in clinical routine, motivated us to explore the feasibility of deriving global and regional cortical thickness measures from contrast‐enhanced MRI. We hypothesized that CE images are a challenge for processing with Freesurfer, resulting in an increased runtime and degraded quality of the reconstructed surface as indicated by the number of surface holes (Monereo‐Sánchez et al., 2021).

In this study, we propose an extension for DL+DiReCT (Rebsamen et al., 2020) to cope with contrast‐enhanced input data by enriching training data of the deep learning (DL) based segmentation model. For the evaluation, we processed a longitudinal dataset of same‐session MR image pairs (non‐enhanced and CE T1w) of MS patients with FreeSurfer and DL+DiReCT and assessed the goodness of the cortical thickness measures. Besides correlation coefficients, we analyzed group differences in annual atrophy rates and metrics from structural covariance networks.

## 2 MATERIALS AND METHODS

### 2.1 MRI dataset

The dataset comprised 454 same‐session pairs of non‐enhanced and contrast‐enhanced (CE), high‐resolution T1w‐MRI from 75 patients with MS. Among them were nine patients with primary progressive MS (PPMS) and 66 with relapsing remitting MS (RRMS). Detailed demographics are reported in Supplementary Table S1.

Patients with RRMS were all under treatment with Natalizumab (Tysabri, Biogen Corp., Cambridge MA, USA), and therefore underwent regular MRI examinations at an interval of ~6 months due to their increased risk of progressive multifocal leukoencephalopathy (PML) (Wattjes & Barkhof, 2014). PPMS patients were examined on an annual interval on average. All images were acquired at the Bern University Hospital (Inselspital) on 1.5 T and 3 T scanners from Siemens (Siemens, Erlangen, Germany) with an MP‐RAGE protocol (Mugler III & Brookeman, 1990; van der Kouwe et al., 2008). Owing to the retrospective nature of the images acquired in clinical routine, sequence parameters were variable as detailed in Supplementary Figure S1. Contrast‐enhanced images were acquired using an MP‐RAGE sequence with a water excitation pulse (Norbeck et al., 2020) after administration of a contrast agent. Patients received an intravenous bolus injection of gadobutrol (0.1 ml/kg body weight Gadovist® 1 mmol/ml, Bayer) or, in case of contraindications, gadoterate meglumine (0.2 ml/kg DOTAREM® 0.5 mmol/ml, Guerbet).

### 2.2 MRI processing

#### 2.2.1 DL+DiReCT

DL+DiReCT is an existing morphometry tool to derive global and regional volumes and cortical thickness measures from T1w MRI (Rebsamen et al., 2020). Its main processing steps include a deep‐learning (DL) based segmentation of tissue classes and cortical parcellations followed by a diffeomorphic registration of the GM/WM boundary to the pial surface to derive a voxel‐wise cortical thickness map (DiReCT) (Das et al., 2009; Tustison et al., 2013).

A new model for the segmentation was added to DL+DiReCT by enriching the training data with CE images. Initially, DL+DiReCT was trained using a total of 840 non‐enhanced T1w MRI, among them 128 images of patients with MS, and auxiliary labels from FreeSurfer, as detailed in Rebsamen et al. (2020). These training data were now enriched with the corresponding 128 same‐session CE MRI of these MS patients. Tissue class labels for the CE images were derived from the non‐enhanced images by co‐registering the image pairs with an affine transformation using *FSL flirt* (Jenkinson et al., 2002; Jenkinson & Smith, 2001) with six degrees of freedom and mutual information as cost function. The resulting transformation matrix was used to resample the CE image into the space of the non‐enhanced image using b‐spline interpolation. Compared to the initial model with 96 labels, separate labels for WM‐hypointensities, left/right inferior horn of the lateral ventricle and choroid plexus were added, resulting in a total of 101 labels. An additional model parcellating the cortex into 74 region per hemisphere according the *Destrieux* atlas (Destrieux et al., 2010) leads to 181 labels. Otherwise, these models were re‐trained with identical hyperparameters and network architecture (McKinley et al., 2019).

The tool is publicly available (https://github.com/SCAN-NRAD/DL-DiReCT) including the new models with the “‐‐model v6” option for the *Desikan‐Killiany* (Desikan et al., 2006) and “‐‐model v7” for the *Destrieux* atlas (Destrieux et al., 2010). Regional cortical thickness and GM volume estimates for the evaluation were generated from the original T1w images. After brain extraction using HD‐BET (Isensee et al., 2019), the new model of DL+DiReCT was applied using default settings (running on one GPU and four CPU cores on Linux).

#### 2.2.2 FreeSurfer (FS)

All MRI were processed with FreeSurfer 6.0 (Fischl, 2012) using the recon‐all pipeline with default settings without manual interventions. Global and regional mean cortical thickness values were extracted from the surface statistics (lh.aparc.stats, rh.aparc.stats) for regions of interest (ROI) as defined by the Desikan‐Killiany atlas (Desikan et al., 2006).

### 2.3 Evaluation

For the evaluation, we processed all non‐enhanced and CE images with FreeSurfer and DL+DiReCT as described above and extracted global and regional mean cortical thickness measures. Pearson correlation coefficients (*r*) were calculated between image pairs, both across all pairs (*n* = 454) as well as on the subset of pairs with identical sequence parameters (TI/TR, *n* = 213). To allow direct comparison with previous work (Lie et al., 2022), intra‐class correlation coefficients (ICC) were calculated based on mean‐rating (*k* = 2), consistency, 2‐way mixed‐effects model (Koo & Li, 2016) with the R‐package *irr* (Gamer et al., 2012).

Leveraging the longitudinal nature of the data, we calculated annual atrophy rates in mm/year by fitting a linear model to the measures of all time points for each patient with age as the covariate. Patients with less than three time points were discarded, resulting in rates from *n* = 73 patients (eight PPMS and 65 RRMS). Group differences in the atrophy rates between the two MS cohorts were quantified with effects sizes using Cohen's *d* (Torchiano, 2019).

Finally, structural covariance networks (SCN) were constructed (Evans, 2013) using the “brain connectivity toolbox” for Python (Rubinov & Sporns, 2010). ROI‐wise cortical thickness measures were corrected for age by fitting linear models to each measure with age as co‐variate. The resulting residuals were then used to calculate correlation matrices. Binary undirected graphs were derived by thresholding the correlation matrix at 0.01 intervals. Confidence intervals were determined with random sampling by repeatedly constructing SCNs from 80% of the images 1000 times. Given multiple sclerosis has been associated with SCN disruption (Tewarie et al., 2014), and since this work is not intended as a complete network study, we focused on the example of *global efficiency* (Latora & Marchiori, 2001) as a metric of network integration to compare the two subgroups of MS patients. In contrast to the mean shortest path length, the global efficiency has a finite value also for disconnected graphs. Based on very recent findings in (Fleischer et al., 2019; Tur et al., 2020) we speculated that SCNs could be differently affected in PPMS and RRMS.

Statistical analyses were performed using *R* with the *stats* package version 3.6.2 (R Core Team, 2019). A significance level *α* = 0.05 was used.

## 3 RESULTS

The median processing runtime of FreeSurfer was 9.3 h (range: 5.8–38.9) for the non‐enhanced images and 15.6 h (7.3–86.3) for the CE images. For DL+DiReCT, the runtimes were 0.3 h (0.2–0.5) for both image types. The median number of surface holes as calculated by FreeSurfer was 74 (range: 15–441) on the non‐enhanced images and 332 (72–1091) on the CE images. Detailed runtime statistics are reported in Supplementary Figures S2 and S3. FreeSurfer failed to process 17 CE images. Subsequently reported results are based on the available images (i.e., based on *n* = 454 images from DL+DiReCT and *n* = 437 from FreeSurfer).

**FIGURE 1:**
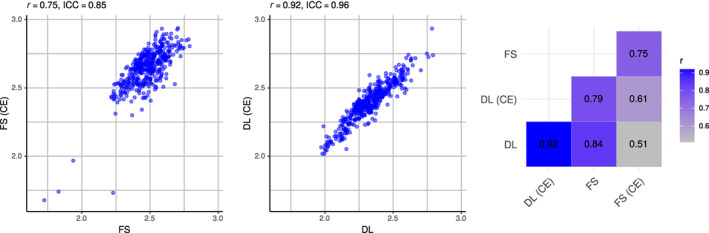
Correlations of global mean cortical thickness measures between all pairs of non‐enhanced (*x*‐axis) and contrast‐enhanced (CE, *y*‐axis) images. Figures for FreeSurfer (FS, left), DL+DiReCT (DL, middle) and combinations thereof (right matrix)

**FIGURE 2:**
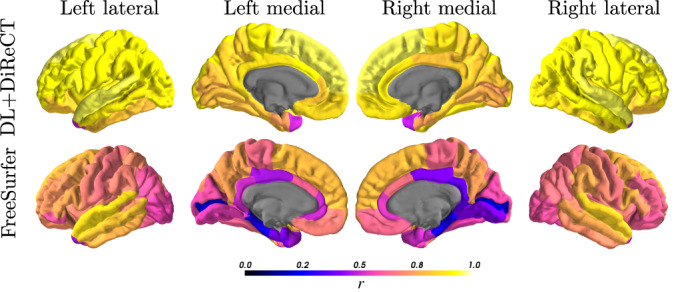
Color‐coded Pearson correlation coefficients (*r*) of the ROI‐wise average cortical thicknesses between measures derived from all pairs of non‐enhanced and CE images

Correlation coefficients for the global mean thickness between all non‐enhanced and CE image pairs were higher for DL+DiReCT (*r* = 0.92 / ICC = 0.96) than for FreeSurfer (*r* = 0.75 / ICC = 0.85) as depicted in Figure 1. Equivalent results were observed for the subset of image pairs with identical MR sequence parameters (DL+DiReCT *r* = 0.96 / ICC = 0.98, FreeSurfer r = 0.87 / ICC = 0.93). When calculated from the contrast‐enhanced images, cortical thickness values were systematically elevated with both methods. This effect was three times more pronounced with FreeSurfer (mean difference = 0.17 mm) than with DL+DiReCT (0.06 mm) as shown in the Bland–Altman plots in Supplementary Figure S4. Regional correlations are depicted in Figure 2, with corresponding values reported in Supplementary Tables S2 and S3 including results for subcortical volumes. Complementary results for the finer‐grained parcellations can be found in Supplementary Figure S5.

**FIGURE 3:**
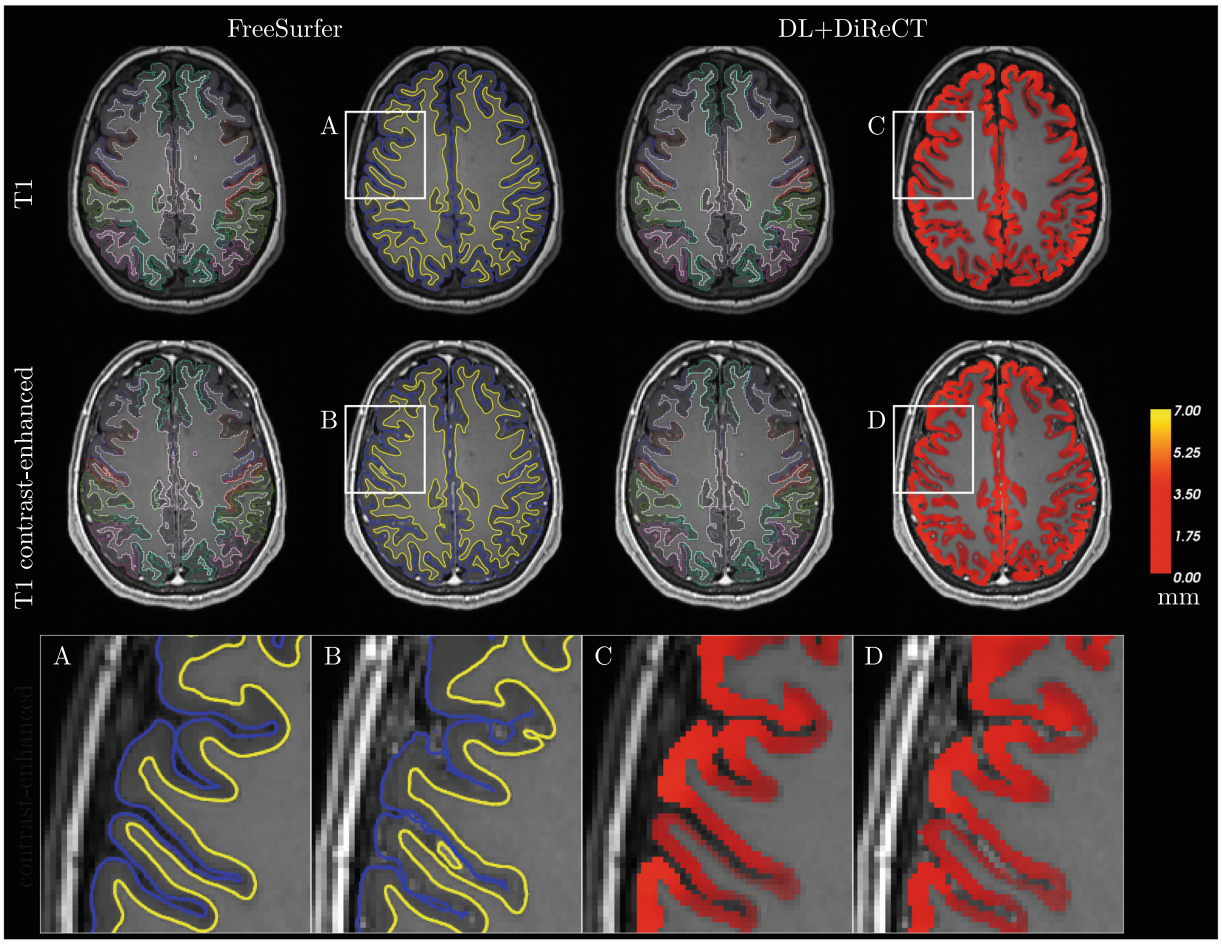
Qualitative example (10%‐quantile subject‐wise correlations across ROIs from DL+DiReCT) with colorized cortical parcellations. To illustrate the cortial thickness, the reconstructed surfaces from FreeSurfer are shown in blue (pial) and yellow (GM/WM) whereas for DL+DiReCT the voxel‐wise thickness map is shown. The magnified view shows distortion artifacts in the FreeSurfer surface from CE image (B), particularly in the sulci. Subject‐wise correlations over all ROIs of the Desikan‐Killiany atlas are *r* = 0.65 for FreeSurfer and *r* = 0.95 for DL+DiReCT (as highlighted in Supplementary Figure S6)

Subject‐wise correlation coefficients (calculated across all 64 regions per subject) were also significantly higher for DL+DiReCT (Supplementary Figure S6). A qualitative example is shown in Figure 3, which was chosen as the subject equal to the 10% quantile from DL+DiReCT, meaning that 410/454 image pairs showed higher and 44/454 showed lower correlation. Similar examples corresponding to the median correlation coefficient and an analysis of an *outlier* are shown in Supplementary Figures S7 and S8.

### 3.1 Annual atrophy rates

**FIGURE 4:**
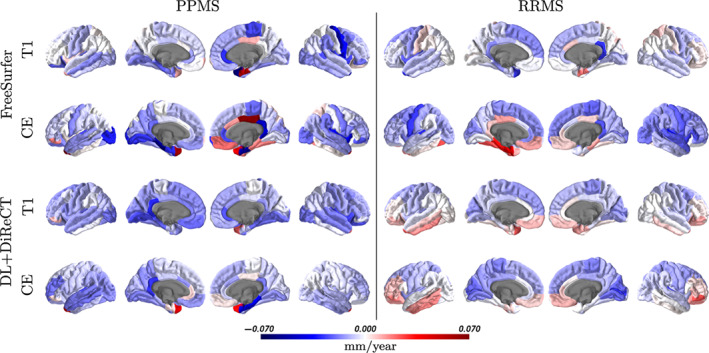
Mean annual regional atrophy rates in mm/year for the two MS subroups derived from non‐enhanced and contrast‐enhanced (CE) images with FreeSurfer and DL+DiReCT

Both methods revealed an increased mean global annual atrophy rates for PPMS patients compared to the RRMS group, except FreeSurfer from CE images (see Table 1). The effect sizes were more pronounced with DL+DiReCT (non‐enhanced = −0.304, CE = ‐0.169) than FreeSurfer (non‐enhanced = −0.111, CE = 0.085). While these group effects were consistently larger than the differences between the two image types for DL+DiReCT, the group effects from FreeSurfer were similar or smaller in magnitude than between the image types (cf. columns *d* vs. last row in Table 1). Increased atrophy rates were also observed regionally for the PPMS group (Figure 4).

**TABLE 1:** Mean annual global atrophy rates (mm/year)

	DL+DiReCT			FreeSurfer		
	Non‐enhanced	CE	*d*	Non‐enhanced	CE	*d*
PPMS [mm/year]	**−0.012**	**−0.009**	**−0.157**	**−0.008**	**−0.009**	**0.084**
RRMS [mm/year]	**−0.003**	**−0.005**	**0.075**	**−0.005**	**−0.011**	**0.224**
Effect size [Cohens'd]	−0.304	−0.169		−0.111	0.085	

*Note*: Effect size between PPMS and RRMS (last row) and between non‐enhanced and CE (column *d*). For better readability, atrophy rates are bold faced.

### 3.2 Structural covariance networks (SCN)

**FIGURE 5:**
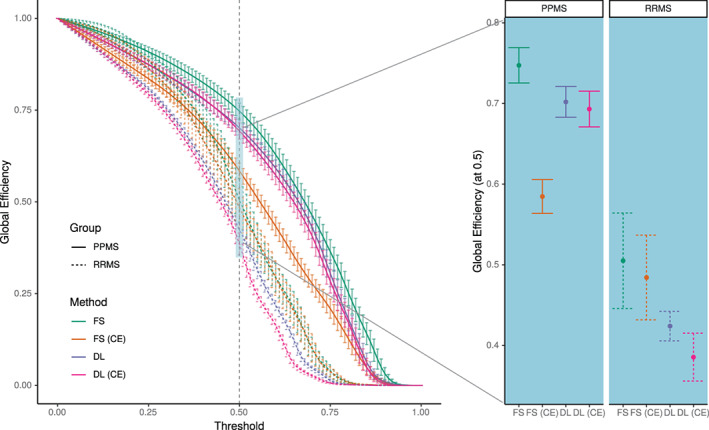
Global efficiency metric for the PPMS and RRMS subgroups calculated from structural covariance networks derived from non‐enhanced and contrast‐enhanced (CE) images with FreeSurfer (FS) and DL+DiReCT (DL). Left: Global efficiency as a function of threshold applied on the correlation matrix. Right: Results corresponding to the threshold at 0.5. Error bars indicate one standard deviation

Analysis using SCN revealed group differences, reflecting a higher global efficiency metric for the PPMS group compared to RRMS (Figure 5). This observation was present for the networks constructed with DL+DiReCT from both image types, as well as with FreeSurfer from the non‐enhanced images. In contrast, no such difference could be observed when calculated with FreeSurfer from the CE images. Confidence intervals of the measures from DL+DiReCT were generally smaller than from FreeSurfer, as indicated for the networks constructed at a threshold of 0.5 (see Figure 5 right).

## 4 DISCUSSION

In this study, we investigated the utilization of contrast‐enhanced (CE) T1‐weighted MRI to derive brain morphometry. A cohort of 75 MS patients with a total of 454 image pairs of non‐enhanced and CE images was used for the evaluation. Results from a baseline generated with FreeSurfer were compared to an extended version of DL+DiReCT by enriching the training data of the deep learning‐based segmentation.

After retraining the model with pairs of non‐enhanced and CE images of MS patients, DL+DiReCT yielded high correlations between cortical thickness and GM volume values calculated from both image types. This was observed, both globally (*r* = 0.91) and regionally. Although FreeSurfer processed most of the post‐contrast images without premature termination (with the exception of 17/454 images, i.e., 3.7%), we observed a substantially higher number of surface holes (Euler number) than for non‐enhanced images. The Euler number can serve as a proxy for the input image quality (Kaufmann et al., 2019) and higher values are generally considered an indicator for a lower quality of the reconstructed surface (Monereo‐Sánchez et al., 2021). Consequently, the correlations of cortical thickness estimates between non‐enhanced and the corresponding CE images were weaker (*r* = 0.75).

Previous work on morphometry from contrast‐enhanced T1w MRI is rare, especially for global and regional cortical thickness measures. A recent dedicated analysis from Lie et al. (2022) reported good to excellent results for global mean thickness (ICC >0.96) using FreeSurfer. The study was, however, based on a much smaller sample size of only 22 patients and included only image pairs with identical MR sequence parameters in the analysis. We were unable to observe such high correlations in our data, even in the subset of image pairs with identical acquisition parameters TI and TR (*r* = 0.87/ICC = 0.93 for FreeSurfer). Whether the additional water excitation pulse (Norbeck et al., 2020) in our contrast‐enhanced MP‐RAGE protocol is causing the remaining difference is difficult to judge. In line with the results from Lie et al. (2022), our results using FreeSurfer yielded an identical mean overestimation of 0.17 mm for the global cortical thickness derived from the CE images compared to the non‐enhanced images. This systematic bias was reduced to 0.06 mm using DL+DiReCT.

More pronounced global annual atrophy rates were observed for the PPMS patients compared to the RRMS group under treatment with Natalizumab, in line with expectations (Portaccio et al., 2013; Preziosa et al., 2020). The rates of the RRMS group were similar to previously reported age‐related atrophy of −0.004 mm/year in healthy cohorts (Lemaitre et al., 2012). However, only the results from DL+DiReCT consistently yielded larger effect sizes between those two groups than between the image types, likely due to the lower robustness of FreeSurfer on the CE images. Regional atrophy rates revealed a better consistency of the patterns between the two image types for DL+DiReCT than for FreeSurfer (cf. Figure 4). In the RRMS group, the pattern suggested accelerated atrophy rates fronto‐parietal as compared to temporal regions. For cortical thickness, less reliable measurement has been reported for the cingulate cortex (Kharabian Masouleh et al., 2020; Rebsamen et al., 2020). We attribute the regions with a putative increase of cortical thickness over time to this kind of uncertainties, especially in the results derived with FreeSurfer from CE images and accentuated in the cingulate cortex and temporo‐basal and temporo‐polar regions.

For SCNs, sizable uncertainties for edge weights and derived graph measures have recently been demonstrated, particularly when estimated from cortical thickness using FreeSurfer (Carmon et al., 2020). Our findings of smaller error bars when using DL+DiReCT instead of FreeSurfer together with larger group separation capabilities strongly suggest DL‐based input for SCN estimation as an alternative. Enhanced robustness of DL+DiReCT compared to FreeSurfer has been reported before (Rebsamen et al., 2020, 2022; Rusak et al., 2022). SCN analysis revealed clear separation between the two subgroups of MS, with DL+DiReCT from both non‐enhanced and CE images. Whether this finding generalizes remains to be investigated since our PPMS subgroup was small and literature about network efficiency in various phenotypes of MS is sparse (Fleischer et al., 2019; Tur et al., 2018, 2020).

### 4.1 Limitations

FreeSurfer failed to process 17 contrast‐enhanced MRIs, and we have made no attempts to re‐process these cases after manual interventions. No pre‐processing of CE images was performed either, and we acknowledge that results from FreeSurfer might yield better results after tuning, e.g., by suppressing high voxel intensities. However, it remains questionable if a globally best threshold or similar parameter could be found and debatable if introducing an additional hyper‐parameter is desirable at all. Pre‐processing of CE images would likely remain manual labor that needs to be performed on an individual case basis.

All MR images in the analysis were from chronic MS patients showing some degree of white matter lesions. Nevertheless, we refrained from *lesion filling* as cortical thickness measures derived with FreeSurfer have shown to be unaffected (Biberacher et al., 2016; Guo et al., 2019). Consequently, our performance assessment concentrated on global and regional mean cortical thickness, while subcortical GM volumes are only reported for completeness in the Supplementary Materials.

Reflecting the lower prevalence of primary progressive compared to relapsing remitting MS in the population, the analyzed subgroups are unbalanced. A substantially lower number of patients in the PPMS group with fewer follow‐up MRIs is a limitation of this study. While group differences for the global atrophy rates are distinct and concordant with expectations from the literature (Portaccio et al., 2013; Preziosa et al., 2020), interpretation of regional atrophy patterns requires a critical appraisal of the results.

Due to the retrospective nature of this study and the fact that images were acquired in clinical routine over a period of 9 years including protocol and scanner upgrades, the data consist of gradient‐echo sequences with variations of sequence parameters and sources from different scanners. These variations likely result in lower correlations. However, even when comparing only pairs of MRI with identical parameters (TI/TR), the effect of considerably higher correlations with DL+DiReCT (*r* = 0.96) than for FS (*r* = 0.87) remained.

### 4.2 Outlook

The ability to derive brain morphometry reliably from contrast‐enhanced T1w MRI will make additional data accessible for quantitative analysis. As a consequence, studies on retrospective clinical data might benefit from a larger sample size. In datasets with both non‐enhanced and contrast‐enhanced images, one could run the analysis twice with both image types, increasing statistical power. For future applications of morphometry on individual patients in clinical routine, running an analysis twice might increase confidence in the results.

While correlations between non‐enhanced and CE images were excellent, there remains a systematic bias in their absolute values. Whether mixing measures from non‐enhanced and CE images in the same analysis is feasible, e.g., by applying a correction factor, remains to be investigated.

## 5 CONCLUSIONS

With the proposed deep learning‐based morphometry tool (DL+DiReCT), brain morphometry can be derived reliably from contrast‐enhanced T1‐weighted MRI. The main findings (effect sizes of atrophy rates between groups and network effects from SCN) in the analyzed cohorts were consistent between the non‐enhanced and contrast‐enhanced images.

## ETHICS STATEMENT

Patients were identified from the existing neuroimmunological registry and retrospective image analysis of clinical routine MRIs was performed (KEK‐BE 2017‐01369, KEK‐BE 2016‐02035).

## Supporting information


Appendix S1:

## Data Availability

The morphometry tool is publicly available, including trained models (https://github.com/SCAN-NRAD/DL-DiReCT). MR images of patients used for the evaluation are not readily available. Derived morphometric measures are available from the authors upon reasonable request.
