# Supplementary material for: Reliable brain morphometry from contrast‐enhanced T1w‐MRI in patients with multiple sclerosis
Source: Hum Brain Mapp. 2022 Oct 17;44(3):970–9. doi: 10.1002/hbm.26117 (PMC9875932; doi:10.1002/hbm.26117)
Supplement: Supplementary file 1 — Appendix S1: [file HBM-44-970-s001.pdf]

Supplementary Materials for Reliable Brain Morphometry from Contrast-Enhanced T1w-MRI in Patients with Multiple Sclerosis

Michael Rebsamen[1,2], Richard McKinley[1], Piotr Radojewski[1,3], Maximilian Pistor[4], Christoph Friedli[4], Robert Hoepner[4], Anke Salmen[4], Andrew Chan[4], Mauricio Reyes[5], Franca Wagner[1], Roland Wiest[1,3], and Christian Rummel[1]

[1]Support Center for Advanced Neuroimaging (SCAN), University Institute of Diagnostic and Interventional Neuroradiology, University of Bern, Inselspital, Bern University Hospital, Bern, Switzerland
[2]Graduate School for Cellular and Biomedical Sciences, University of Bern, Bern, Switzerland
[3]Swiss Institute for Translational and Entrepreneurial Medicine, sitem-insel, Bern, Switzerland
[4]Department of Neurology, Inselspital, Bern University Hospital and University of Bern, Bern, Switzerland
[5]ARTORG Center for Biomedical Research, University of Bern, Bern, Switzerland

Group	# MRI (# Subjects)	Mean # follow-up (range)	Mean follow-up period in years (range)	Mean age in years (range)	%Female
PPMS	39 (9)	4.3 (2-7)	4.2 (1.0-7.2)	50.2 (36.1-64.0)	30.8%
RRMS	415 (66)	6.3 (2-9)	2.7 (0.6-5.6)	36.5 (15.8-60.0)	65.3%
Total	454 (75)	6.1 (2-9)	2.9 (0.6-7.2)	37.7 (15.8-64.0)	62.3%

Table S1: Demographic information for the cohorts. Statistics for age and sex are calculated over the MRI samples at the time of acquisition.

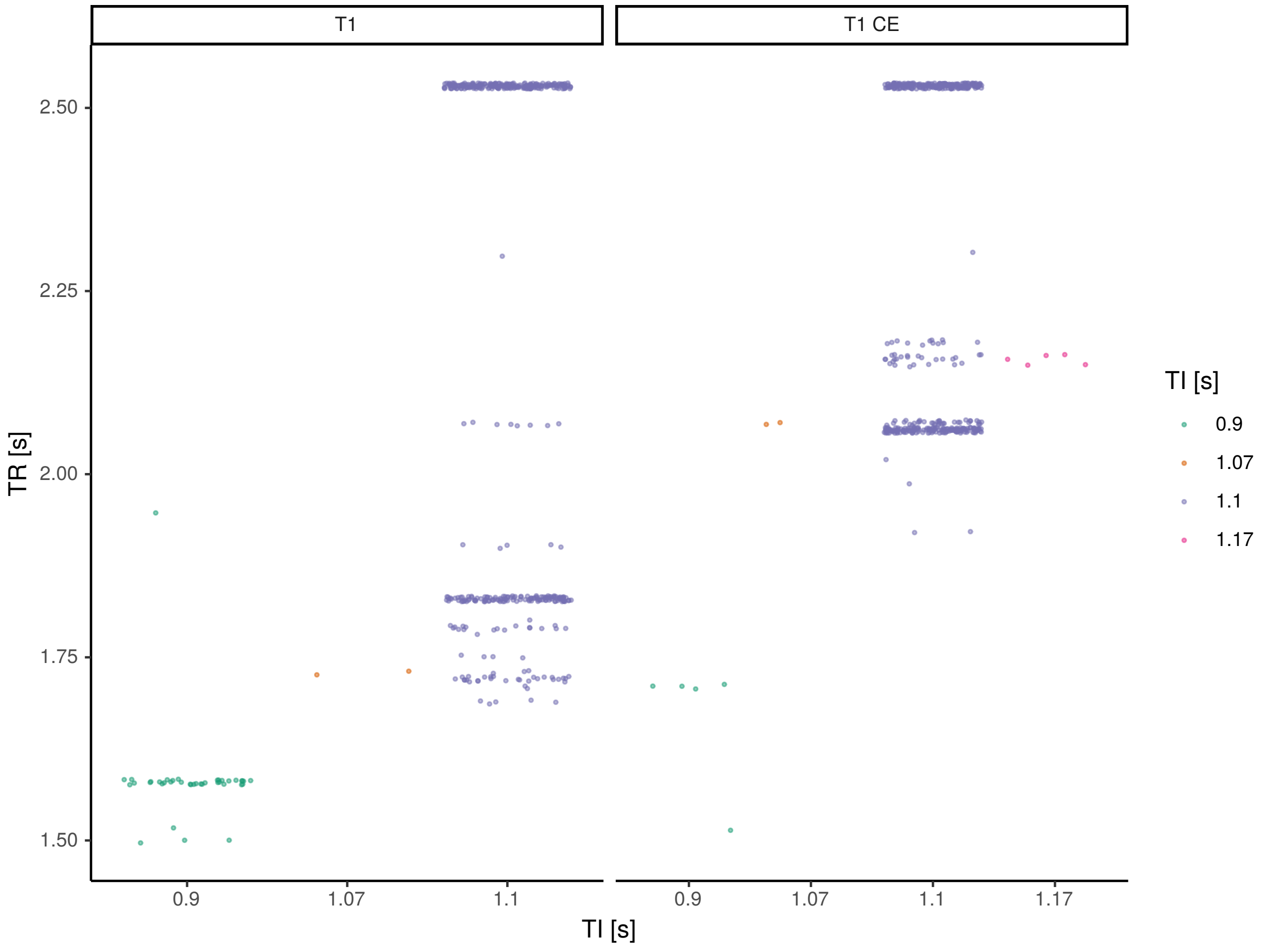

Figure S1: Visualization of the various MR acquisition parameters for the pairs of non-enhanced T1 and contrast-enhanced (CE) images. Repetition time (TR) on the y-axis and inversion time (TI) on the x-axis with jitter for better readibility. The topmost 213 image pairs have identical acquisition parameters (TR=2.53 seconds, TI=1.1 seconds)

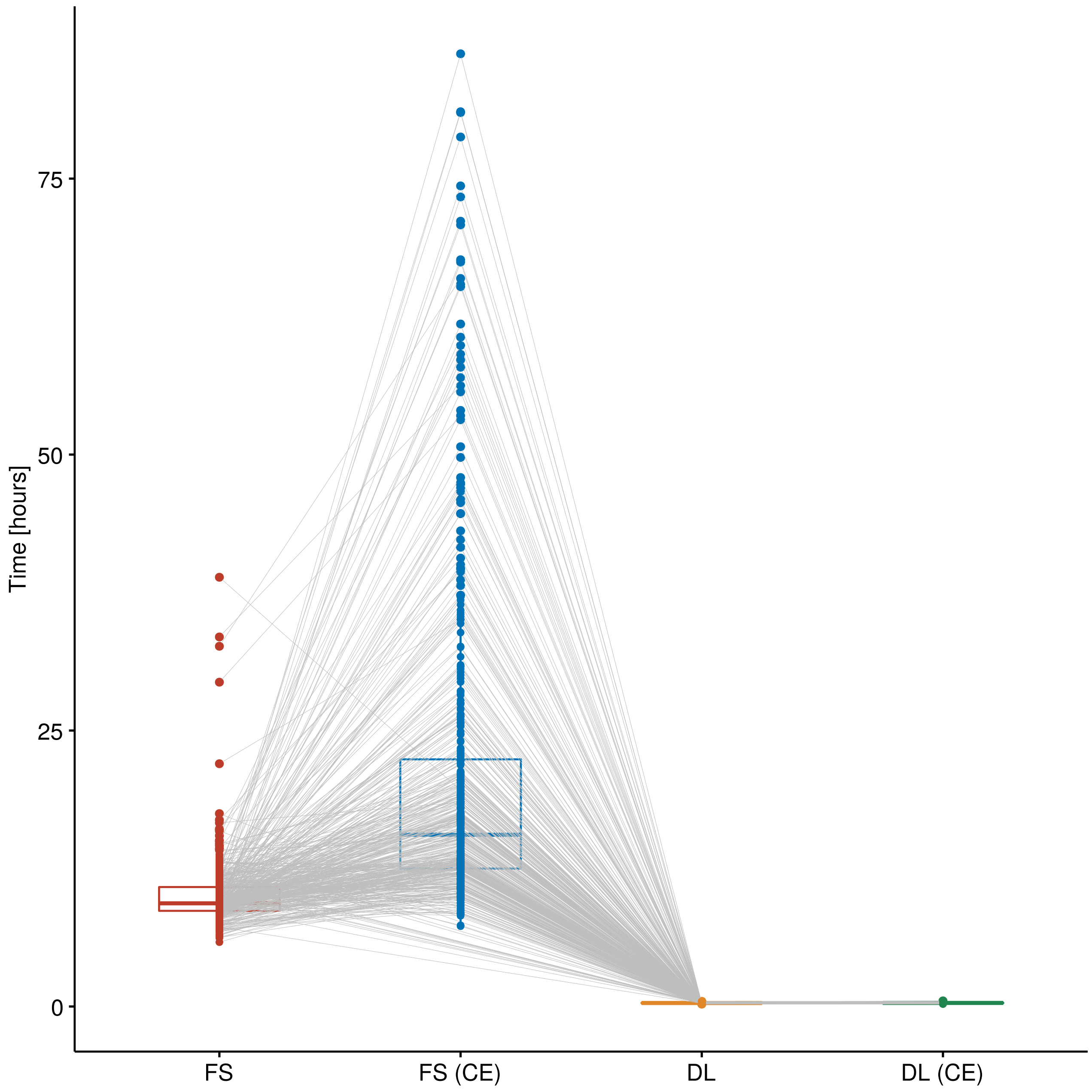

Figure S2: Total processing runtime for non-enhanced and contrast-enhanced (CE) images using FreeSurfer (FS) and DL+DiReCT (DL). Whiskers of the boxplots indicate 1.5× interquartile range. Gray lines connect corresponding non-enhanced and contrast-enhanced images.

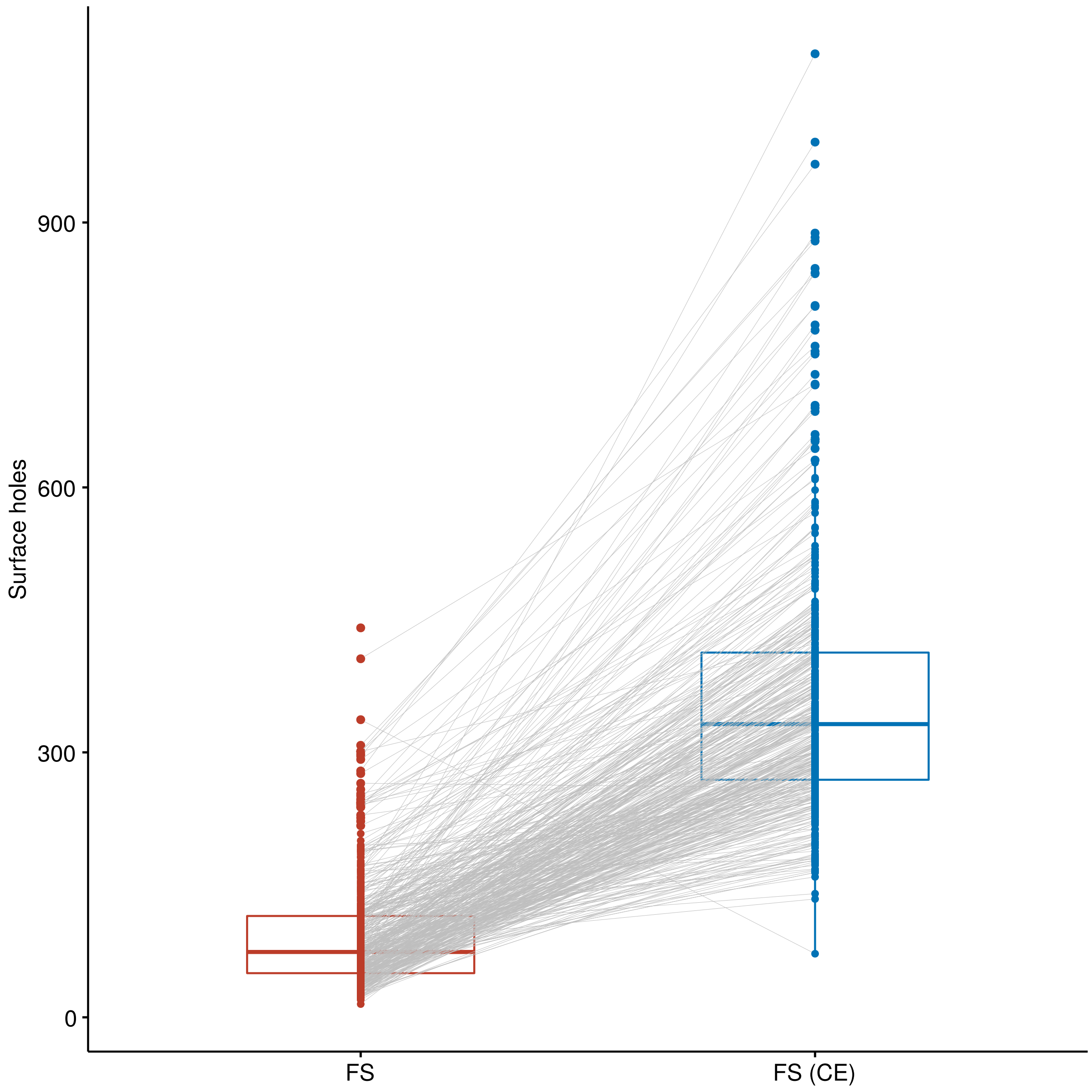

Figure S3: Number of surface holes (Euler number) of the reconstructed cortical surface using FreeSurfer on the non-enhanced (left) and contrast-enhanced (CE, right) images. Whiskers of the boxplots indicate 1.5× interquartile range. Gray lines connect corresponding non-enhanced and contrast-enhanced images.

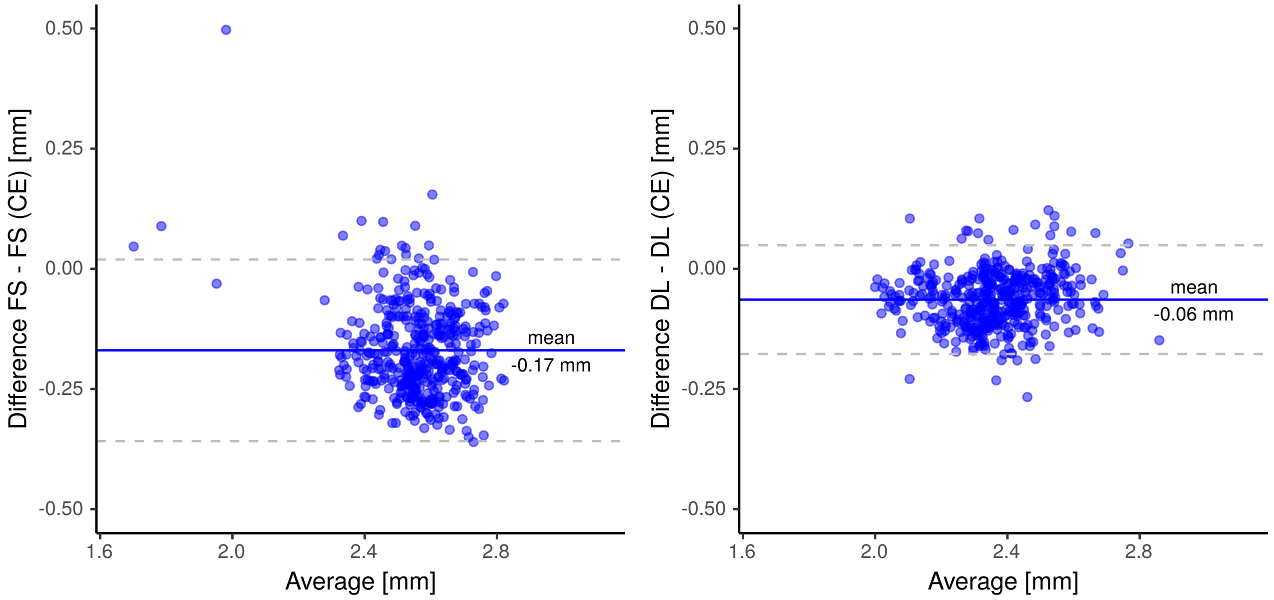

Figure S4: Bland–Altman plots of the global mean thickness derived from the non-enhanced and contrast-enhanced (CE) images for FreeSurfer (left) and DL+DiReCT (right). The difference is plotted on the y-axis against the average on the x-axis. Dashed gray lines represent 95% confidence intervals.

		FreeSurfer		FreeSurfer (CE)		DL+DiReCT		DL+DiReCT (CE)	
		lh	rh	lh	rh	lh	rh	lh	rh
Cortical Thickness [mm]	Banks of the sup. temp. sulcus	2.48±0.19	2.57±0.21	2.71±0.21	2.75±0.23	1.98±0.24	1.98±0.19	2.06±0.29	2.05±0.20
	Caudal ant. cingulate cortex	2.70±0.24	2.57±0.25	2.59±0.24	2.45±0.26	2.47±0.32	2.60±0.27	2.49±0.33	2.68±0.25
	Caudal middle frontal gyrus	2.53±0.21	2.51±0.21	2.78±0.22	2.77±0.20	2.14±0.21	2.13±0.22	2.15±0.20	2.15±0.20
	Cuneus	1.83±0.14	1.87±0.14	2.01±0.19	2.02±0.18	1.59±0.17	1.61±0.17	1.64±0.17	1.71±0.19
	Entorhinal cortex	3.29±0.33	3.35±0.40	3.15±0.38	3.23±0.43	3.32±0.36	3.39±0.40	3.37±0.35	3.46±0.36
	Frontal pole	2.80±0.29	2.77±0.29	3.02±0.35	3.06±0.34	2.97±0.32	3.03±0.32	3.03±0.32	3.13±0.35
	Fusiform gyrus	2.71±0.17	2.73±0.18	2.79±0.17	2.80±0.19	2.89±0.20	2.99±0.22	3.11±0.22	3.23±0.26
	Inferior parietal lobule	2.44±0.16	2.49±0.16	2.72±0.17	2.75±0.17	2.21±0.18	2.29±0.18	2.21±0.18	2.33±0.18
	Inferior temporal gyrus	2.79±0.18	2.79±0.17	2.99±0.20	2.97±0.19	3.06±0.26	3.17±0.27	3.21±0.23	3.37±0.27
	Insular cortex	2.98±0.21	2.98±0.21	3.05±0.23	3.04±0.22	2.83±0.19	2.84±0.18	2.92±0.19	2.92±0.18
	Isthmus cingulate cortex	2.40±0.21	2.44±0.21	2.22±0.27	2.19±0.32	2.55±0.22	2.53±0.23	2.61±0.27	2.65±0.28
	Lateral occipital cortex	2.11±0.14	2.19±0.13	2.44±0.24	2.50±0.23	2.09±0.18	2.18±0.19	2.17±0.20	2.30±0.21
	Lateral orbitofrontal cortex	2.67±0.17	2.64±0.18	3.00±0.21	2.98±0.21	2.72±0.20	2.73±0.21	2.81±0.20	2.84±0.21
	Lingual gyrus	1.99±0.14	2.02±0.13	2.19±0.21	2.19±0.19	1.98±0.19	2.03±0.18	2.22±0.25	2.23±0.24
	Medial orbitofrontal cortex	2.48±0.17	2.47±0.16	2.65±0.20	2.71±0.18	2.61±0.24	2.58±0.25	2.85±0.24	2.83±0.27
	Middle temporal gyrus	2.84±0.21	2.88±0.18	3.02±0.21	3.08±0.19	2.94±0.25	2.93±0.22	3.00±0.24	3.02±0.24
	Paracentral lobule	2.31±0.19	2.35±0.18	2.28±0.23	2.36±0.23	1.85±0.20	1.88±0.17	1.83±0.18	1.89±0.16
	Parahippocampal gyrus	2.80±0.29	2.74±0.25	2.36±0.31	2.41±0.28	2.37±0.29	2.46±0.28	2.57±0.31	2.73±0.32
	Pars opercularis	2.58±0.20	2.59±0.21	2.80±0.22	2.81±0.21	2.28±0.21	2.24±0.22	2.32±0.20	2.31±0.22
	Pars orbitalis	2.77±0.21	2.77±0.23	3.09±0.28	3.08±0.28	2.81±0.24	2.86±0.24	2.82±0.26	2.92±0.28
	Pars triangularis	2.51±0.20	2.49±0.18	2.82±0.24	2.79±0.21	2.24±0.21	2.27±0.21	2.25±0.19	2.31±0.21
	Pericalcerine cortex	1.56±0.13	1.58±0.13	1.67±0.19	1.66±0.18	1.14±0.14	1.16±0.14	1.17±0.15	1.23±0.17
	Postcentral gyrus	2.09±0.15	2.05±0.15	2.24±0.14	2.21±0.15	1.74±0.14	1.66±0.14	1.73±0.13	1.70±0.14
	Posterior cingulate cortex	2.48±0.17	2.50±0.17	2.31±0.23	2.32±0.24	2.30±0.19	2.39±0.19	2.36±0.23	2.46±0.23
	Precentral gyrus	2.49±0.19	2.46±0.20	2.58±0.23	2.56±0.21	2.04±0.18	2.00±0.18	2.02±0.16	2.01±0.17
	Precuneus	2.37±0.15	2.38±0.15	2.49±0.17	2.52±0.18	2.10±0.16	2.12±0.15	2.15±0.15	2.19±0.15
	Rostral ant. cingulate cortex	2.86±0.21	2.89±0.23	2.82±0.22	2.83±0.24	3.01±0.24	2.95±0.29	3.02±0.25	3.06±0.28
	Rostral middle frontal cortex	2.41±0.16	2.37±0.17	2.70±0.19	2.67±0.18	2.33±0.23	2.31±0.22	2.37±0.22	2.40±0.23
	Superior frontal gyrus	2.69±0.19	2.68±0.20	2.83±0.19	2.85±0.20	2.48±0.22	2.47±0.22	2.50±0.20	2.52±0.21
	Superior parietal lobule	2.19±0.15	2.17±0.14	2.41±0.16	2.39±0.16	1.85±0.15	1.83±0.15	1.86±0.14	1.88±0.15
	Superior temporal gyrus	2.78±0.21	2.82±0.20	2.90±0.22	2.98±0.21	2.49±0.23	2.45±0.20	2.50±0.22	2.49±0.20
	Supramarginal gyrus	2.54±0.17	2.54±0.17	2.76±0.17	2.76±0.18	2.33±0.19	2.31±0.20	2.34±0.17	2.36±0.19
	Temporal pole	3.57±0.33	3.67±0.38	3.48±0.43	3.62±0.40	3.88±0.45	4.05±0.42	4.07±0.42	4.20±0.44
	Transverse temporal gyrus	2.39±0.26	2.44±0.27	2.29±0.28	2.30±0.28	2.04±0.27	2.07±0.26	2.16±0.29	2.23±0.26
	**Global mean thickness**	2.47±0.13	2.47±0.13	2.64±0.15	2.65±0.14	2.33±0.15	2.34±0.15	2.38±0.14	2.41±0.15
Subcortical Volumes [ml]	Amygdala	1.49±0.22	1.64±0.22	1.47±0.21	1.64±0.20	1.58±0.19	1.63±0.19	1.62±0.19	1.68±0.20
	Caudate nucleus	3.25±0.43	3.33±0.47	2.96±0.40	2.98±0.43	3.34±0.43	3.44±0.47	3.20±0.42	3.32±0.47
	Cerebral white matter	222.09±27.54	221.35±27.57	221.31±28.43	221.00±28.20	215.38±28.04	215.73±27.94	215.26±28.13	215.01±28.01
	Choroid plexus	0.66±0.24	0.66±0.23	0.37±0.21	0.37±0.23	0.47±0.15	0.48±0.16	0.42±0.14	0.42±0.15
	Globus pallidus	1.85±0.28	1.83±0.26	1.85±0.25	1.84±0.25	1.83±0.26	1.81±0.26	1.79±0.27	1.78±0.28
	Hippocampus	3.87±0.40	3.96±0.43	3.94±0.74	4.03±0.79	4.20±0.41	4.24±0.40	4.22±0.42	4.20±0.40
	Inf. horn of lateral ventricle	0.46±0.31	0.48±0.32	0.31±0.28	0.36±0.25	0.39±0.29	0.46±0.30	0.35±0.28	0.45±0.29
	Lateral ventricle	12.36±6.75	11.04±6.09	10.66±5.92	9.61±5.45	12.49±6.97	11.14±6.33	12.56±6.92	11.28±6.25
	Nucleus accumbens	0.40±0.09	0.45±0.09	0.44±0.10	0.48±0.09	0.45±0.09	0.42±0.08	0.38±0.09	0.36±0.08
	Putamen	4.19±0.66	4.28±0.70	4.16±0.64	4.20±0.66	4.33±0.65	4.36±0.64	4.20±0.67	4.22±0.67
	Thalamus	6.90±0.97	6.65±0.91	8.65±1.46	7.88±1.28	6.73±0.91	6.94±0.91	6.75±0.91	6.91±0.88
	Ventral diencephalon	3.83±0.47	3.74±0.46	4.46±0.61	4.44±0.57	4.08±0.41	4.04±0.42	4.07±0.44	4.02±0.44
	Brainstem	20.79±2.86		20.53±2.91		20.23±2.63		19.70±2.62	
	White matter hypointensities	4.04±4.07		5.02±4.61		3.24±3.55		3.09±3.67	

Table S2: Cohort-wide mean±standard deviation of regional and global measures for left (lh) and right hemisphere (rh), derived from the non-enhanced T1 images followed by the corresponding measures from the contrast-enhanced (CE) images. Note: standard deviations represent distributions across the cohorts and do not reflect the error of the measures.

		FreeSurfer		DL+DiReCT		FS vs. DL	
		lh	rh	lh	rh	lh	rh
Cortical Thickness	Banks of the sup. temp. sulcus	0.718	0.704	0.909	0.910	0.547	0.666
	Caudal ant. cingulate cortex	0.619	0.655	0.914	0.873	0.331	0.225
	Caudal middle frontal gyrus	0.751	0.764	0.919	0.920	0.883	0.899
	Cuneus	0.586	0.576	0.795	0.809	0.621	0.644
	Entorhinal cortex	0.415	0.514	0.735	0.744	0.608	0.649
	Frontal pole	0.757	0.702	0.866	0.847	0.771	0.669
	Fusiform gyrus	0.624	0.555	0.789	0.874	0.429	0.480
	Inferior parietal lobule	0.604	0.657	0.921	0.903	0.816	0.751
	Inferior temporal gyrus	0.767	0.704	0.808	0.855	0.461	0.575
	Insular cortex	0.691	0.677	0.884	0.904	0.554	0.677
	Isthmus cingulate cortex	0.514	0.382	0.869	0.827	0.134	0.203
	Lateral occipital cortex	0.564	0.597	0.893	0.876	0.489	0.551
	Lateral orbitofrontal cortex	0.711	0.622	0.814	0.799	0.636	0.685
	Lingual gyrus	0.552	0.422	0.795	0.766	0.504	0.586
	Medial orbitofrontal cortex	0.679	0.634	0.853	0.879	0.653	0.562
	Middle temporal gyrus	0.810	0.755	0.889	0.922	0.496	0.646
	Paracentral lobule	0.642	0.639	0.897	0.870	0.855	0.749
	Parahippocampal gyrus	0.280	0.280	0.807	0.820	0.735	0.674
	Pars opercularis	0.749	0.780	0.928	0.924	0.729	0.787
	Pars orbitalis	0.598	0.608	0.815	0.837	0.676	0.604
	Pars triangularis	0.744	0.719	0.911	0.906	0.837	0.780
	Pericalcerine cortex	0.215	0.235	0.771	0.734	0.441	0.323
	Postcentral gyrus	0.656	0.697	0.903	0.924	0.767	0.771
	Posterior cingulate cortex	0.467	0.422	0.873	0.807	0.251	0.299
	Precentral gyrus	0.684	0.663	0.912	0.906	0.841	0.840
	Precuneus	0.743	0.739	0.825	0.818	0.750	0.734
	Rostral ant. cingulate cortex	0.509	0.539	0.814	0.890	0.627	0.457
	Rostral middle frontal cortex	0.719	0.632	0.914	0.907	0.776	0.870
	Superior frontal gyrus	0.787	0.792	0.946	0.947	0.897	0.890
	Superior parietal lobule	0.707	0.641	0.911	0.903	0.889	0.845
	Superior temporal gyrus	0.860	0.838	0.942	0.944	0.746	0.783
	Supramarginal gyrus	0.686	0.755	0.901	0.932	0.705	0.795
	Temporal pole	0.470	0.525	0.503	0.476	0.472	0.509
	Transverse temporal gyrus	0.673	0.638	0.870	0.843	0.704	0.714
	**Global mean thickness**	0.751	0.734	0.918	0.924	0.828	0.844
Subcortical Volumes	Amygdala	0.795	0.776	0.882	0.927	0.767	0.820
	Caudate nucleus	0.938	0.936	0.977	0.984	0.945	0.940
	Cerebral white matter	0.947	0.950	0.993	0.993	0.930	0.928
	Choroid plexus	0.693	0.687	0.791	0.837	0.769	0.793
	Globus pallidus	0.777	0.768	0.946	0.936	0.784	0.722
	Hippocampus	0.417	0.391	0.912	0.914	0.889	0.863
	Inf. horn of lateral ventricle	0.928	0.944	0.984	0.982	0.949	0.939
	Lateral ventricle	0.996	0.997	0.999	0.999	0.997	0.998
	Nucleus accumbens	0.768	0.868	0.894	0.914	0.602	0.702
	Putamen	0.883	0.911	0.979	0.980	0.921	0.946
	Thalamus	0.301	0.277	0.988	0.987	0.874	0.890
	Ventral diencephalon	0.689	0.670	0.976	0.972	0.897	0.891
	Brainstem	0.919		0.980		0.954	
	White matter hypointensities	0.934		0.994		0.976	

Table S3: Pearson correlation coefficients (r) between non-enhanced and contrast-enhanced image pairs, for left (lh) and right hemisphere (rh). As reference, the correlations between the measurres from FreeSurfer (FS) and DL+DiReCT (DL) derived from the non-enhanced images are shown in the last two columns, similar to Figure 3 in the original publication (Rebsamen et al., 2020).

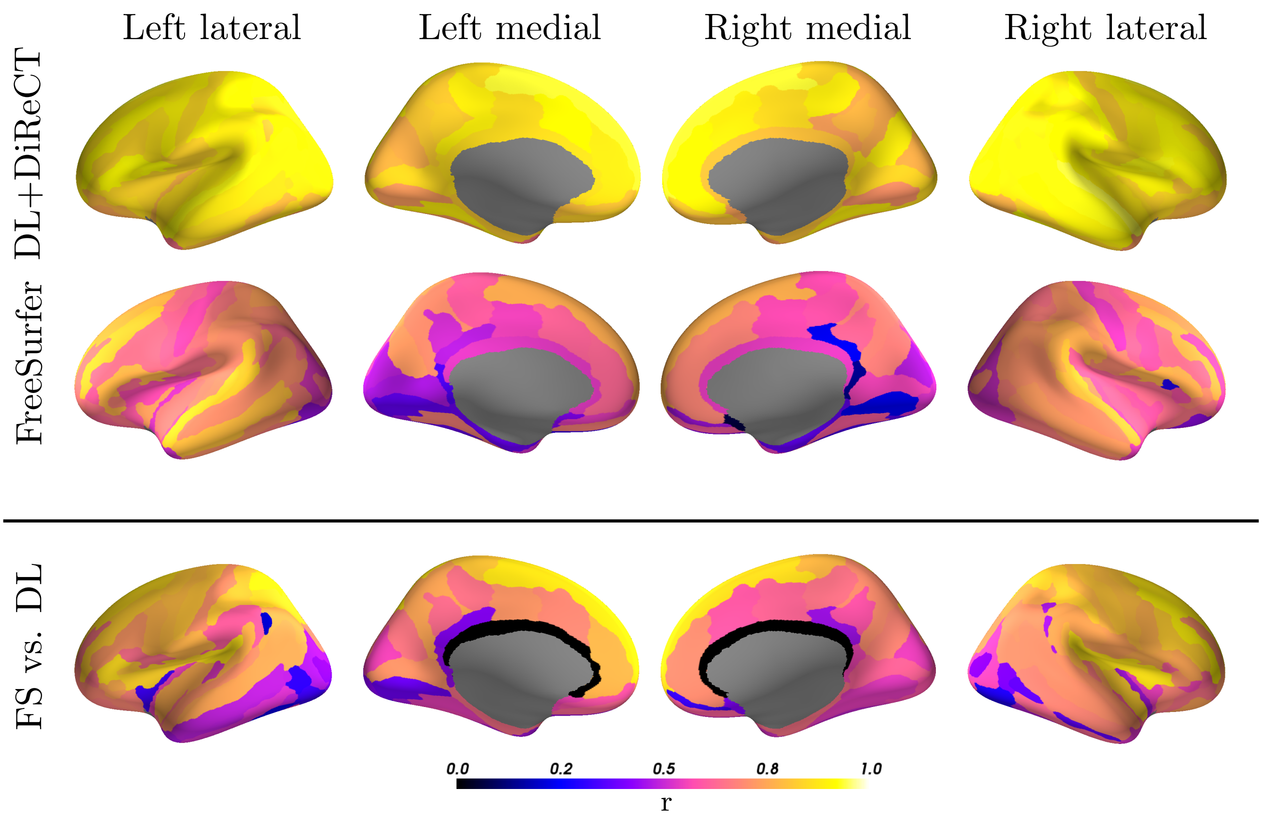

Figure S5: Color-coded Pearson correlation coefficients (r) of the ROI-wise average cortical thickness estimates for the 74 parcellations per hemisphere of the *Destrieux* atlas. First two rows show correlations between measures derived from all pairs of non-enhanced and CE images for DL+DiReCT and FreeSurfer. As a reference below, the correlations between measures from FreeSurfer (FS) and DL+DiReCT (DL) derived from the non-enhanced images is shown.

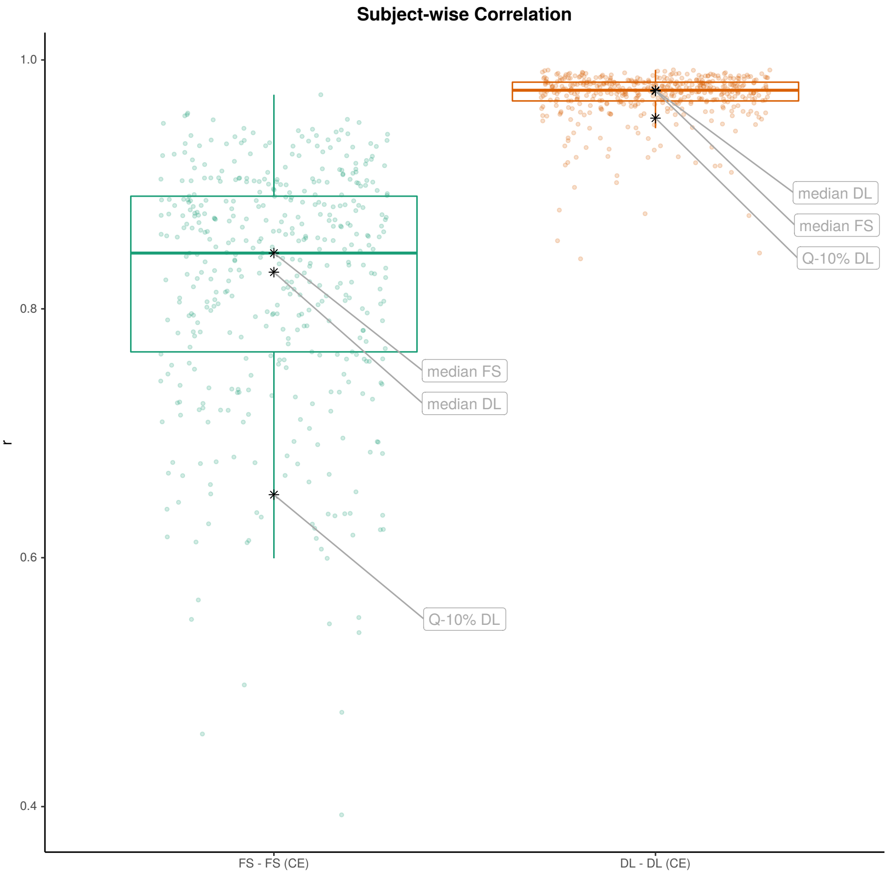

Figure S6: Correlation coefficient calculated for every MR image pair calculated across all 64 regional cortical thickness measures. FS: FreeSurfer, DL: DL+DiReCT, CE: contrast-enhanced. The qualitative results of the highlighted 10%-quantile of DL+DiReCT is shown in the main text and the median of DL+DiReCT (DL) and FreeSurfer (FS) in the Supplementary Material. Whiskers of the boxplots indicate 1.5× interquartile range.

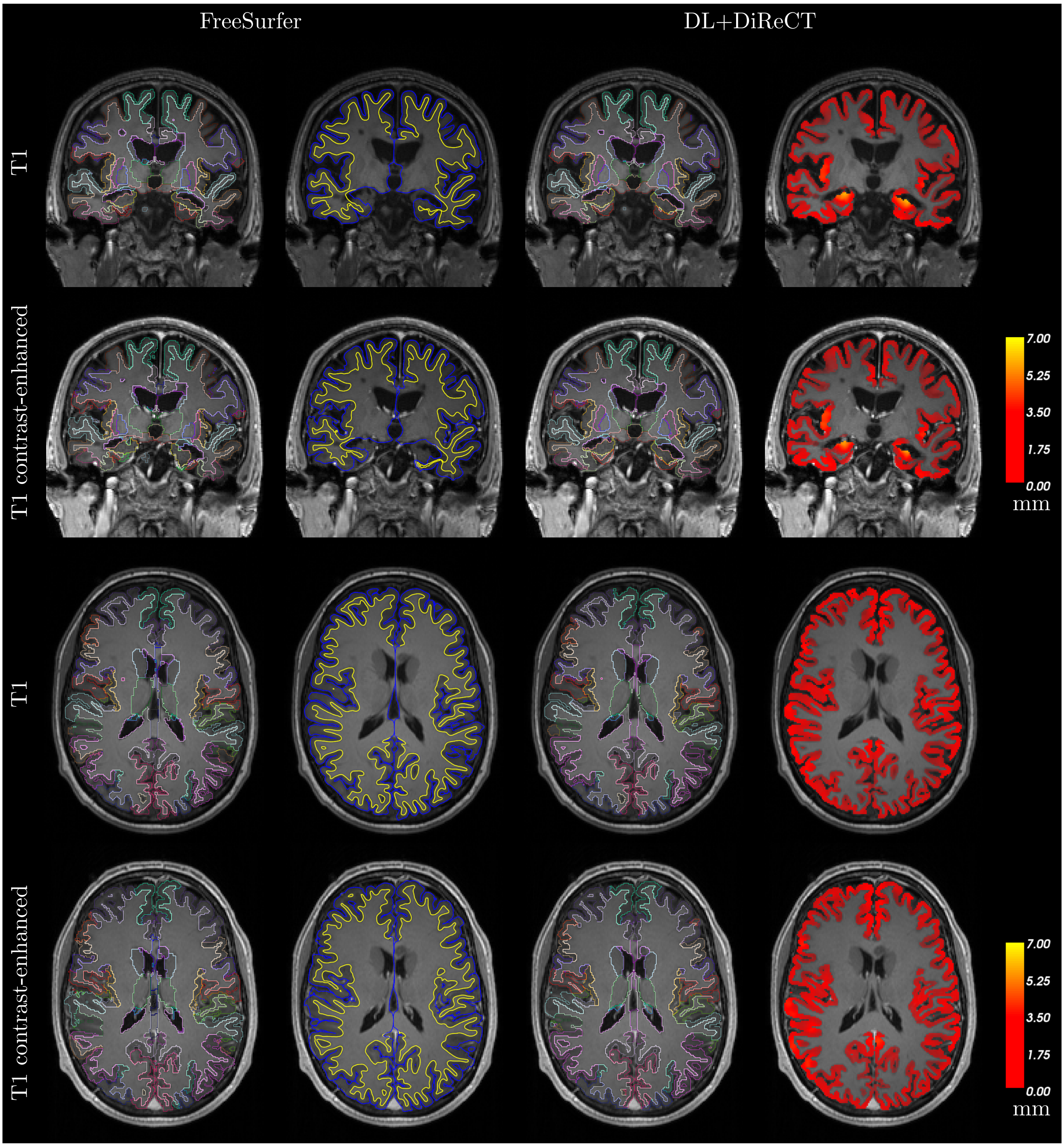

Figure S7: Two qualitative examples with colorized cortical parcellations. For FreeSurfer, the reconstructed surfaces are shown in blue (pial) and yellow (GM/WM) whereas for DL+DiReCT the voxel-wise thickness map is shown. The examples were chosen by the median subject-wise correlation between cortical thickness calculated from non-enhanced and CE images across regions for DL+DiReCT (top images shown in coronal view, $r = 0.829$ for FreeSurfer and $r = 0.976$ for DL+DiReCT) and for FreeSurfer (bottom images shown in axial view, $r = 0.845$ for FreeSurfer and $r = 0.975$ for DL+DiReCT).

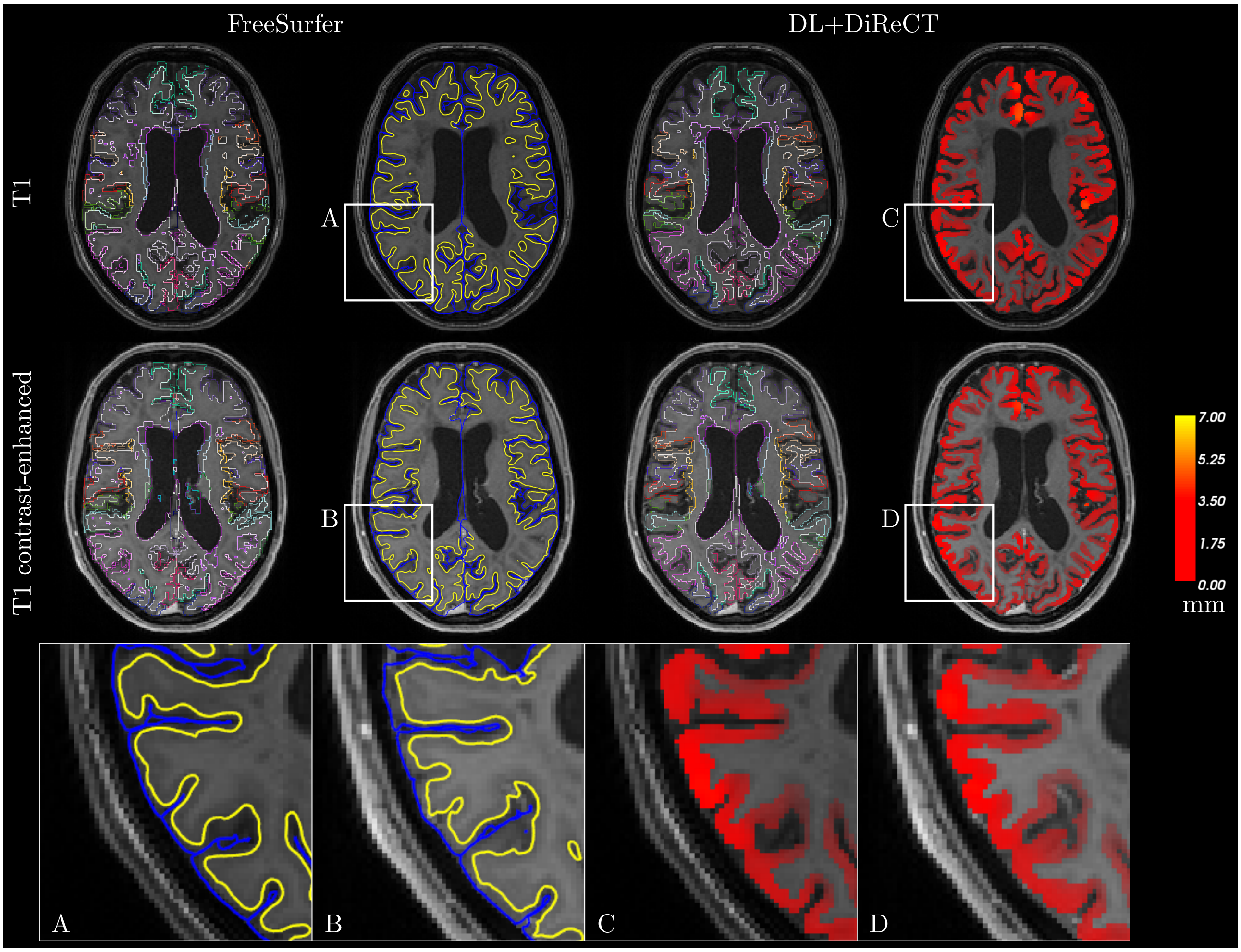

Figure S8: Qualitative example of an *outlier* case with the thinnest cortex measured with FreeSurfer (cf. Figure 1 in the manuscript). All four cases with a global mean thickness below 2 mm measured on the contrast-enhanced MRI with FreeSurfer belong to the same individual: A 32-year old female patient with RRMS. The high lesion load, early onset of MS at the age of 24 and the severity of the disease with an EDSS of 8 likely caused the severly pronounced atrophy. However, FreeSurfer substantially underestimated the cortical thickness due to a misplacement of the GM/WM boundary (in yellow, see magnified section *A* and *B*). Global mean thickness measures from FreeSurfer were 1.72 mm and 1.68 mm from the non-enhanced and contrast-enhanced images respectively, and 2.16 mm and 2.05 mm from DL+DiReCT.
